# Whole genome sequencing and prediction of antimicrobial susceptibilities in non-tuberculous mycobacteria

**DOI:** 10.3389/fmicb.2022.1044515

**Published:** 2022-11-29

**Authors:** Priya Solanki, Marc Lipman, Timothy D. McHugh, Giovanni Satta

**Affiliations:** ^1^UCL-TB and UCL Centre for Clinical Microbiology, University College London, London, United Kingdom; ^2^UCL-TB and UCL Respiratory, University College London, London, United Kingdom; ^3^Royal Free London NHS Foundation Trust, London, United Kingdom

**Keywords:** *Mycobacterium*, non-tuberculous mycobacteria, resistance, sequencing, mutations

## Abstract

Non-tuberculous mycobacteria (NTM) are opportunistic pathogens commonly causing chronic, pulmonary disease which is notoriously hard to treat. Current treatment for NTM infections involves at least three active drugs (including one macrolide: clarithromycin or azithromycin) over 12 months or longer. At present there are limited phenotypic *in vitro* drug susceptibility testing options for NTM which are standardised globally. As seen with tuberculosis, whole genome sequencing has the potential to transform drug susceptibility testing in NTM, by utilising a genotypic approach. The Comprehensive Resistance Prediction for Tuberculosis is a database used to predict *Mycobacterium tuberculosis* resistance: at present there are no similar databases available to accurately predict NTM resistance. Recent studies have shown concordance between phenotypic and genotypic NTM resistance results. To benefit from the advantages of whole genome sequencing, further advances in resistance prediction need to take place, as well as there being better information on novel drug mutations and an understanding of the impact of whole genome sequencing on NTM treatment outcomes.

## Introduction

Non-tuberculous mycobacteria (NTM) include over 160 *Mycobacterium* species (other than *Mycobacterium tuberculosis* and *Mycobacterium leprae*) and are found world-wide typically in soil and water (including municipal supplies) (Ratnatunga et al., 2020). NTM are classified into two broad microbiological groups: rapid growing (RGM) and slow growing (SGM) NTM based on the time it takes for colonies to form on solid subculture. Rapid growing NTM, e.g., *Mycobacterium abscessus*, take < 7 days to form colonies whereas slow growing NTM are usually ≥ 7 days (Sharma and Upadhyay, 2020). NTM are opportunistic pathogens which most frequently are associated with chronic, pulmonary disease that is notoriously hard to treat (Schiff et al., 2019). Pulmonary disease is largely found in immunocompromised patients or patients with pre-existing lung conditions, such as cystic fibrosis (CF), bronchiectasis or chronic obstructive pulmonary disease (COPD) (Wu et al., 2018). Although there is some global variation, overall *Mycobacterium avium* complex (MAC) (which includes *M. avium, M. intracellulare*, and *M. chimaera*) and *M. abscessus* account for up to 90% of the total number of reported cases of pulmonary NTM disease (Lipman et al., 2020). Recurrent infections with MAC are frequently attributed to a re-infection, whereas recurrent infections with *M. abscessus* complex are linked to treatment failure (Nishiuchi et al., 2017; Weng et al., 2020). Non-pulmonary diseases associated with NTM are often presented as skin and soft tissue infections which are usually caused by *Mycobacterium marinum, M. chimaera*, and *M. abscessus* (Johansen et al., 2020).

The prevalence of NTM infections is increasing globally, which in turn has resulted in a heightened awareness of the clinical relevance of these infections (Lipman et al., 2020). The incidence of NTM infections has shown to have increased over the last two decades in North America, Europe, and Asia. Epidemiological data from the UK shows the average annual prevalence of NTM infections was 6.38 per 100,000 between 2006 and 2016, and 27.7 per 100,000 in patients with NTM associated respiratory disease (Axson et al., 2018). In 2013, the USA prevalence rose from 2.4 cases/100,000 in the early 1980s to 15.2 cases/100,000 (Ratnatunga et al., 2020). Recent evidence also suggests the possibility of person-to-person transmission in *M. abscessus*, although further research efforts are required, which emphasises the increasing relevance of these mycobacterial infections (Lipworth et al., 2021). Treatment choices for NTM are largely based on professional opinion and case series rather than randomly controlled trials. This (limited) evidence base underpins the British Thoracic Society (BTS) and American Thoracic Society/Infectious Diseases Society of America (ATS/IDSA) guidelines (Griffith et al., 2007; Daley et al., 2020). Currently, treatment of NTM infections involves at least three active drugs (including one macrolide: clarithromycin or azithromycin) over 12 months or longer (Lange et al., 2022). Several factors are considered when deciding treatment options, e.g., the need for specific antimicrobial therapy depending on the signs and symptoms of the patient, the specific organism and the presumed mycobacterial load (i.e., if a larger bacterial load, more drugs are likely to be added to the regimen). Lengthy treatment regimens for NTM infections result in similar treatment timeframes as multi-drug resistant *M. tuberculosis*, both of which at times use multiple drugs with often uncertain efficacy (Chen et al., 2019). Despite the use of several drugs over a long period of time the treatment outcome remains inadequate with significant adverse effects (Wu et al., 2018). MAC infections causing pulmonary disease have particularly poor treatment outcomes with a 60% rate of treatment success, defined as either culture negativity for 12 months while on therapy or achievement of culture conversion and treatment completion without relapse (Kwak et al., 2017). *M. abscessus* complex infections, caused by *M. abscessus subsp. abscessus* are particularly difficult to treat due to at least 80% of isolates being macrolide resistant (van Ingen et al., 2013). At present there are limited phenotypic *in vitro* drug susceptibility testing (DST) options for NTM, which have been standardised globally and international guidelines only make recommendations on the DST testing of few drugs (macrolides, amikacin and rifampicin, depending on the NTM considered) (Griffith et al., 2007; Daley et al., 2020). Susceptibility test-based treatments are limited by several factors: (i) poor correlation between *in vitro* drug sensitivity results and clinical outcomes (especially for *M. abscessus*); (ii) differences in the *in vitro* and *in vivo* conditions; (iii) natural resistance present in some NTM; (iv) variants with variant specific drug sensitivity profiles; (v) absence of large-scale clinical trials for testing novel treatment regimens. (Brown-Elliott and Woods, 2019; Chen et al., 2019; Maitra et al., 2021). In a post-genomic era, this review discusses the potential of whole genome sequencing (WGS) to predict antimicrobial susceptibilities in NTM and enable more focused treatment with reduced adverse events.

## Current methods for drug susceptibility testing in non-tuberculous mycobacteria

The most widely used DST method in diagnostic laboratories is the broth microdilution method, which is recommended by the Clinical Laboratory Standards Institute Guidelines (CLSI) (Huang et al., 2020). Broth microdilution is used to determine the minimum inhibitory concentration (MIC), defined as the lowest concentration of antibiotic required to inhibit visible growth, using concentrations derived from serial twofold dilutions. As with all commercial DST assays, broth microdilution requires positive mycobacterial cultures to initiate the DST. Conditions for incubation differ between RGM and SGM within CLSI in that SGM plates are incubated at 37°C for 7–14 days whereas RGM are incubated at 30 ± 2°C for 2–5 days. The media used is cation adjusted Mueller Hinton broth (CAMBH). Variations in the protocol may also be required for some NTM, e.g., *M. xenopi* require incubation at 42°C and *M. abscessus* plates should be further incubated and checked at day 7, 10, and 14 in order to detect inducible resistance to macrolides such as clarithromycin (discussed later) (Rampacci et al., 2020).

Indirect colorimetric DST assays such the Resazurin assay (REMA) and Alamar blue, in which the metabolic activity of bacteria changes the colour of the indicator reagent, require enriched 7H9 media as opposed to CAMHB utilised in CLSI microdilution methods (Rampacci et al., 2020). Other DST methods to determine SGM MIC include agar proportion and the use of the BACTEC Mycobacteria Growth Indicator Tube (MGIT) 960 (Becton Dickinson). However, these methods involve complicated inoculum calibration and frequently result in inconsistent results due to interspecies variability and intraspecies differences (Rampacci et al., 2020).

### *Mycobacterium tuberculosis* as a paradigm for whole genome sequencing susceptibilities

Whole genome sequencing has already demonstrated its value in a diagnostic setting for *M. tuberculosis*, with England’s national reference laboratories being the first in the world to use WGS for both the diagnosis and detection of drug resistance in *M. tuberculosis* (Satta et al., 2018). As WGS has replaced phenotypic testing for the prediction of first-line *M. tuberculosis* drugs, in England, this has led to a potential pathway for individualised treatment approaches globally (Dookie et al., 2022). A recent review found a 20% increase in the detection of drug resistance compared to established genotypic assays such as the GeneXpert MTB/RIF (Cepheid, USA) and the GenoType MTBDRplus (v2.0) (Hain Lifescience, Germany) (Lam et al., 2021).

In 2018, the World Health Organization (WHO) published a technical guide on the use of WGS for the detection of mutations associated with drug resistant *M. tuberculosis*. This concluded that WGS might be regarded as a more robust reference standard for defining certain resistance profiles, for example, resistance to Pyrazinamide (PZA) which is challenging to test phenotypically (World Health Organization, 2018). The most common sequencing platform used for *M. tuberculosis* is Illumina MiSeq (Illumina) which has the advantage of high throughput and high sequence yield with relatively low costs and low input DNA (Dookie et al., 2022).

Whole genome sequencing reports, including resistance data in *M. tuberculosis*, can be generated in 24 days (from sample collection) compared to at least 29 days for conventional susceptibility methods (Pankhurst et al., 2016), and so it can be expected that WGS would have similar benefits in lowering the turnaround time (TAT) for NTM susceptibility results. However, a recent report from a clinical microbiology laboratory has shown their TAT from sample collection to WGS was 34 days as opposed to 24 days in 2016 (Park et al., 2022).

## Mutations associated with drug resistance in non-tuberculous mycobacteria

The Comprehensive Antibiotic Resistance Database (CARD) is a collection of well-characterised, peer-reviewed resistance determinants and their associated antibiotics. Genes and mutations conferring drug resistance in NTM can be found using the CARD, where users are able to download the gene sequences and compare these to WGS data obtained (Alcock et al., 2020). An initial search for “*Mycobacterium*” contained 100 results of which 77 results were associated with *M. tuberculosis* resistance and 5 results for NTM—relating to *M. intracellulare, M. avium, Mycobacterium kansasii* (*M. kansasii*), and *M. abscessus*. A further search for “*Mycobacteroides*,” resulted in 12 resistance mutations associated with *M. abscessus and Mycobacterium chelonae* (*M. chelonae*). Both *Mycobacterium* and *Mycobacteroides* were searched due to the current debate in genus names (Oren and Trujillo, 2019). See Table 1.

**TABLE 1:** Mutations found from a comprehensive antibiotic resistance database search for NTM, including the relevant gene and its reference.

CARD search for “Mycobacterium”			
NTM	Gene	CARD short name	References
*Mycobacterium intracellulare*	23S (ribosomal Ribonucleic acid) rRNA with mutation conferring resistance to azithromycin	Mint_23S_AZM	Meier et al., 1994
	23S rRNA with mutation conferring resistance to clarithromycin	Mint_23S_CLR	Meier et al., 1994
*Mycobacterium avium*	23S rRNA with mutation conferring resistance to clarithromycin	Mavi_23S_CLR	Nash and Inderlied, 1995
*Mycobacterium kansasii*	23S rRNA with mutation conferring resistance to clarithromycin	Mkan_23S_CLR	Burman et al., 1998
*Mycobacterium abscessus*	Class A beta-lactamase	MAB	Soroka et al., 2014; Lefebvre et al., 2017
**CARD search for “Mycobacteroides”**			
*Mycobacteroides abscessus*	16S rRNA mutation conferring resistance to kanamycin	Mabs_16S_KAN	Prammananan et al., 1998; Nessar et al., 2011
	16S rRNA mutation conferring resistance to neomycin	Mabs_16S_NEO	Prammananan et al., 1998
	16S rRNA mutation conferring resistance to gentamicin	Mabs_16S_GEN	Prammananan et al., 1998; Nessar et al., 2011
	16S rRNA mutation conferring resistance to tobramycin	Mabs_16S_TOB	Prammananan et al., 1998; Nessar et al., 2011
	23S rRNA with mutation conferring resistance to clarithromycin	Mabs_23S_CLR	Wallace et al., 1996; Mougari et al., 2017
	16S rRNA mutation conferring resistance to amikacin	Mabs_16S_AMK	Prammananan et al., 1998; Nessar et al., 2011
*Mycobacteroides chelonae*	16S rRNA mutation conferring resistance to kanamycin A	Mche_16S_KAN	Prammananan et al., 1998
	16S rRNA mutation conferring resistance to neomycin	Mche_16S_NEO	Prammananan et al., 1998
	16S rRNA mutation conferring resistance to gentamicin C	Mche_16S_GENC	Prammananan et al., 1998
	16S rRNA mutation conferring resistance to tobramycin	Mche_16S_TOB	Prammananan et al., 1998
	23S rRNA with mutation conferring resistance to clarithromycin	Mche_23S_CLR	Vester and Douthwaite, 2001
	16S rRNA mutation conferring resistance to amikacin	Mche_16S_AMK	Prammananan et al., 1998
*Mycolicibacterium smegmatis*	23S rRNA with mutation conferring resistance to clarithromycin	Msme_23S_CLR	Sander et al., 1997

Searches for “clarithromycin” and ‘amikacin” within the database are highlighted in light grey and dark grey, respectively.

Alternatively, a search for “clarithromycin” (the most administered first line drug for NTM) obtained six results for: *M. abscessus, M. chelonae, M. kansasii, M. avium, M. intracellulare*, and *M. smegmatis* (highlighted light grey in Table 1). A CARD search for “amikacin” had one result for *M. abscessus* and one for *M. chelonae* (highlighted dark grey in Table 1).

Additionally, a PubMed search for “sequencing resistance non-tuberculous mycobacteria” resulted in 622 articles (search date 01 August 2022). A filter was applied to only include articles from the last 10 years and resulted in 390 articles of which 93 contained information on genetic mutations in NTM (most commonly referring to clarithromycin and amikacin) that could lead to drug resistance. These are summarised below. Other databases, e.g., PATRIC, are able to provide genes and mutations conferring drug resistance in NTM. When investigated further, PATRIC has inaccuracies in the references noted and is unable to provide information on *M. abscessus* (Davis et al., 2020). We have focused on *M. abscessus* as it is currently a clinically topical NTM due to being intrinsically multi-drug resistant and thus a challenge where rapid drug susceptibility results are required in order to optimise patient management (Saxena et al., 2021). Resistance to clarithromycin in RGM, occurring most commonly in the *M. abscessus* complex (MABC), arises *via* two mechanisms: acquired resistance which involves spontaneous point mutations; and methylation resulting in inducible resistance, requiring the presence of a functional erythromycin ribosome methylase *erm*(*41*) gene (Rubio et al., 2015; Victoria et al., 2021).

Clarithromycin mutations associated with acquired resistance are found in the *rrl* gene, encoding for 23S rRNA, at the 2058 or 2059 adenine positions (Meier et al., 1994). Should these mutations be found using WGS, it would suggest phenotypic resistance to Clarithromycin. Isolates with these mutations are observed to be resistant to clarithromycin during the day 3–5 DST readings. Inducible resistance occurs in the presence of clarithromycin, or other macrolides, when the *erm*(*41*) gene is expressed. The methylase produced transfers one or two methyl groups to A2058 in the peptidyl transferase region of the 23S rRNA, i.e., the drug target (Degiacomi et al., 2019). This results in clarithromycin being unable to bind to the ribosome. Inducible resistance can be detected phenotypically by extending the incubation time of the DST. Broth microdilution plates are checked at days 7, 10, and 14 for inducible resistance, which is defined as detected growth at clarithromycin concentrations ≥ 8 μg/ml (CLSI, 2011).

The *M. abscessus* complex (MABC) consists of three subspecies: *M. abscessus*, *M. boletti*, and *M. massiliense*. *M. abscessus* and *M. boletti* possesses a complete *erm*(*41*) gene conferring resistance to clarithromycin (Nessar et al., 2011). In contrast, *M. massiliense* as well as *M. chelonae* have an absent or truncated *erm*(*41*) gene and are therefore likely to be sensitive to clarithromycin and other macrolides (de Carvalho et al., 2018; Brown-Elliott and Woods, 2019). A T/C polymorphism at the 28th nucleotide (T28C) (shown by WGS) enables reversion to susceptibility, as T28 MABC strains show inducible resistance whilst C28 strains are susceptible (Degiacomi et al., 2019).

Resistance in amikacin, as well as other aminoglycosides, is the result of the modification of the 30S ribosomal subunit (Degiacomi et al., 2019). The key 16S rRNA (*rrs*) gene mutations are in 1406, 1408, 1409, 1411, or 1491 base pairs (Brown-Elliott and Woods, 2019). Should any one of these spontaneous mutations be noted *via* WGS then the isolate is likely to be amikacin resistant. However, there may still be unknown mechanisms of amikacin resistance, and as such the absence of 16S rRNA mutations does not imply amikacin sensitivity (Prammananan et al., 1998; Brown-Elliott and Woods, 2019). This would also be in line with *M. tuberculosis*, where other mutations (*eis* promoter and *rpsL*) can confer resistance to the aminoglycosides class (Alangaden et al., 1998).

Repurposed antibiotics, acting against validated targets for other infections, have been gaining interest in the treatment of NTM infections due to the increased prevalence of drug resistance (Gutiérrez et al., 2019). Bedaquiline (BDQ) and clofazimine (CFZ) are currently used to treat multi-drug resistant tuberculosis (TB) and recently, these two antibiotics have been explored in the treatment of NTM pulmonary infections caused by MABC (Ruth et al., 2019). An important resistance marker for CFZ and BDQ in MABC is spontaneous mutations in the DNA binding domain of *MAB_2299c*, which encodes a TetR transcriptional regulator that represses the expression of two MmpS-MmpL efflux pumps encoded by *MAB_2300-MAB_2301* and *MAB_1135c–MAB_1134c* genes. This would have some affinity with the mechanism of resistance described for *M. tuberculosis*, where the *Rv0678* is another transcriptional regulator conferring resistance to BDQ and CFZ and whose frame is located downstream of the *mmpS5-mmpL5* operon (Radhakrishnan et al., 2014; Nimmo et al., 2020). The mutations in the DNA binding domain result in elevated expression of the efflux pumps and thus increased drug efflux levels leading to resistance (Gutiérrez et al., 2019; Johansen et al., 2020). Intrinsic resistance to BDQ and CFZ in *M. avium* and *M. intracellulare* also involves the over expression of efflux pumps (Alexander et al., 2017). Finally, resistance to Imipenem, another first line drug for MABC, is associated with the presence of the *Bla*_*Mab*_ gene encoding a class A β-lactamase. The *Bla*_*Mab*_ gene is similar to the *BlaC* gene of *M. tuberculosis* (Lopeman et al., 2020).

## Discussion

The CLSI recommended broth microdilution DST method has its limitations. Firstly, the concentrations tested are in serial two-fold dilutions where the true MIC could be between any two concentrations and MIC determination can be subject to variation depending on technical experience. It is debatable if such potential variation in MICs has an impact in patients’ outcomes with the standard oral dosing, and it is certainly not relevant for inhaled drugs (i.e., amikacin) where the lung concentration achieved is much higher compared to the laboratory breakpoint (Rubino et al., 2021). Plate incubation conditions necessarily vary between RGM and SGM, which add an additional complexity when isolates with mixed infections of RGM and SGM are not identified prior to DST, e.g., with *M. avium* and MABC mixed infection. There are currently no clear guidelines on how mixed infections are to be treated, i.e., how the SGM or RGM would be treated if both had varied resistance profiles. A fundamental requirement of all DST methods is the need to standardise media to avoid variation in readout as a result of the varied physiological environment. The DST for NTM can use both CAMBH and enriched 7H9 liquid media depending on experimental aims and these have been shown to result in conflicting MICs when all other variables are constant. For example, Nicklas et al. (2022) compared the MIC of omadacycline (a promising oral tetracycline alternative to tigecycline, for NTM treatment) when tested against 32 *M. abscessus* isolates, using both CAMBH and 7H9 broth. The results showed that omadacycline trended lower for the majority of the *M. abscessus* strains in CAMBH compared to the MIC values in 7H9 (Nicklas et al., 2022). Further research demonstrating the clinical importance of conflicting MICs, due to media, would be useful in order to determine whether such variables could impact clinical decision making. A further consideration when novel NTM treatment options are being evaluated is their interaction with the media. For example, clofazimine has been shown to be insoluble in CAMBH and thus requires alternative media (Ferro et al., 2016; Ruth et al., 2019).

Genotypic tests such as line probe assays, e.g., the GenoType NTM-DR test (Hain Lifescience, Germany) are available for both the identification and prediction of clarithromycin and amikacin resistance. However, the detection of resistance within these assays is limited by the mutations chosen (Bouzinbi et al., 2020), whilst WGS can identify other mutations that the line probe assay is unable to detect (Realegeno et al., 2021). See Table 2 for a summary of the advantages and disadvantages of phenotypic DST and WGS, for use in the prediction of antimicrobial susceptibilities in NTM.

**TABLE 2:** Summary of the advantages and disadvantages of phenotypic DST and WGS, for use in the prediction of antimicrobial susceptibilities in NTM.

	Advantages	Disadvantages	Further improvements
Phenotypic DST	● Established method ● Reproducibility ● Direct measurement of susceptibilities ● Widely understood interpretation	● Long TAT ● Varying incubation requirements ● Complicated inoculum calibration ● Inconsistent results ● Subject to variation depending on technical experience ● True MIC could be between any two concentrations ● Poor correlation between *in vitro* drug sensitivity results and clinical outcomes	● Research demonstrating the clinical importance of conflicting MICs ● Evaluating novel drugs with regard to plate media
WGS	● Identifies additional mutations compared to genotypic tests ● Reduction in TAT ● Concordance between phenotypic and WGS results ● Skill set widely available ● Accessible in low and middle-income countries ● Can be used in conjunction with machine learning-without the need for databases of known resistance genes	● Inferred susceptibility results ● Lack of databases available which can accurately predict resistance ● Requires high value equipment and software ● Requires a skilled bioinformatician ● DNA must be extracted from positive cultures and cannot be performed directly from primary samples ● Impact of WGS on NTM treatment outcomes are unknown	● Advancements in the accurate prediction of resistance in NTM *via* databases ● Develop machine learning models for use with NTM ● Identify all mutations associated with novel drugs ● The need for clinical studies describing the value WGS adds on front line decision making ● Investigate the cost-effectiveness

As has been demonstrated for tuberculosis, WGS has the potential to transform DST in NTM, by utilising a genotypic approach to predict drug susceptibility and by avoiding the various DST issues described above. Realegeno et al. (2021) investigated the value of replacing traditional DST with WGS for the prediction of clarithromycin and amikacin resistance in MABC. Clinical samples were investigated, of which 58 MABC isolates were shown to be phenotypically resistant to clarithromycin by broth microdilution. Inducible resistance was seen genotypically in 56/58 isolates by the presence of a full-length *erm*(41), with a T at position 28 and acquired resistance shown in 2/58 isolates based on mutations in the *rrl* gene at position 2270 (A2270T). The results of the study demonstrated 100% concordance between phenotypic and genotypic resistance results (Realegeno et al., 2021). Similarly, amikacin genotypic resistance also showed 100% concordance with phenotypic results by the identification of A-to-G substitutions at position 1408 of the *rrs* gene (Realegeno et al., 2021).

At present, there are no databases available which could accurately predict resistance from WGS data of NTM, matched with DST results. The Comprehensive Resistance Prediction for Tuberculosis (CRyPTIC) is a database, led by the University of Oxford, for *M. tuberculosis*. The initiative aims to provide better WGS assembly to identify more *M. tuberculosis* variants and use enhanced statistical methods that can detect connections between variants and DST. Databases such as CRyPTIC could revolutionise NTM drug resistance identification whilst improving patient management with faster TAT (UKRI Gateway, 2022). Artificial intelligence (AI) methods such as machine learning models, especially deep learning algorithms, are valuable when used in conjunction with WGS to complement the prediction of antimicrobial susceptibilities. Machine learning uses genomic sequences obtained from WGS and encodes the sequences into numerical values, which are subsequently used in the classification of drug susceptibilities (Liu et al., 2022). Ren et al. (2022) investigated machine learning methods in the prediction of drug susceptibilities, with results showing the models used can predict resistance without the need for databases of known resistance genes (Liu et al., 2022; Ren et al., 2022).

Turnaround time for NTM DST results is often inadequate, with reporting times taking up to 2 months (from collection) depending on the NTM tested. The TAT is extended in the case of *M. abscessus* samples when they are sensitive to clarithromycin, as they are required to be incubated for a further 14 days to detect inducible macrolide resistance (Huang et al., 2020). Technical workflows for WGS could be performed in 3–5 days post culture positivity (assuming the DNA obtained is of good quality and quantity) with the data analysis taking a couple of hours. This could significantly reduce the TAT for current susceptibility testing. The reduction in TAT can be considered one of the major advantages of WGS over phenotypic DST (Brown-Elliott and Woods, 2019).

The WHO technical guide on the use of WGS for the detection of mutations associated with drug resistant *M. tuberculosis* concluded, that although WGS might be regarded as a more robust reference standard for defining certain resistance profiles, there are limitations to its use in clinical settings. Firstly, there is limited knowledge on the genetic diversity of *M. tuberculosis* in certain regions. This is also true for clinically significant NTM, and hence a limitation for the use of WGS to predict resistance. Secondly, the studies investigated by WHO did not include all known resistance genes for *M. tuberculosis* drugs and this limits knowledge on rare mutations such as *eis* promoter mutations and amikacin resistance (World Health Organization, 2018). Regarding NTM, the fundamental genetic mutations conferring resistance were determined in the late 90 s (see Table 1) and there is the potential that many rare mutations are yet to be identified.

Although only a few NTM are clinically significant, the genetic variation both between and within species adds additional complexity when creating resistance predicting databases. A further complication to moving to WGS for NTM DST is that novel treatments for NTM have varied genetic markers and it cannot be assumed that known *M. tuberculosis* markers are the same for NTM. Currently identified *M. tuberculosis* genes which confer resistance to BDQ are *atpE*, *Rv0678*, and *pepQ* genes. Although mutations in the *atpE* gene are also associated with BDQ resistance in MABC, there are additional mutations which are specific to the *Mycobacterium* species, e.g., within the *MAB_2299c* gene with others yet to be discovered (Gutiérrez et al., 2019; Johansen et al., 2020).

Current practical challenges of WGS roll out include removing the requirement of high value equipment and obtaining readouts that are useable by scientists/clinicians with minimal bioinformatics experience. At present, the processing of sequence data requires software which may not be widely available and a skilled bioinformatician (Dohál et al., 2021). In addition, DNA must be extracted from positive cultures and the process could be optimised if WGS could be performed directly from primary samples (Dohál et al., 2021). As a result of the existing practical challenges, WGS should initially be used in conjunction with phenotypic DST for maximum data output as the process is still in its infancy and requires considerable development prior to being exclusively used in diagnostic laboratories. From a clinical perspective, the impact of WGS on NTM treatment outcomes still needs to be demonstrated. Park et al. (2022) investigated the clinical impact of routine WGS in tuberculosis treatment decisions. The study found 12/20 patients required changes in treatment due to isoniazid resistance being identified by WGS, demonstrating the added benefit for clinical management (Park et al., 2022).

Mycobacteriology is a specialised field and requires experienced personnel, however the skill set for WGS is more widely available and therefore could be more accessible in low and middle-income countries compared to conventional microbiology (Kekre et al., 2021). In order to benefit from the advantages of WGS, further advancements need to be made in the accurate prediction of resistance in NTM *via* databases, or advancing AI machine learning methods, as well as identifying all mutations associated with novel drugs. Having WGS data widely available *via* an open access platform would aid in the implementation of the Findable, Accessible, Interoperable, and Reusable (FAIR) principles. FAIR enhances the reusability of scientific data by machines which can automatically find data for users. Open research challenges for WGS include; large file sizes which require large capacity hard-drives; the need for globally recognised nomenclature for NTM and agreed quality standards including the acceptable coverage from sequencing (Wilkinson et al., 2016). Furthermore, clinical studies describing the value WGS adds on front line decision making for alterations in drug treatment, as well as investigating cost-effectiveness, will be crucial in evaluating the use of the technology as a whole.

## Author contributions

PS, TM, and GS conceived the original idea of the manuscript. PS wrote the first draft. All authors have reviewed and commented on the final draft.
